# Morphologically Homogeneous Red Blood Cells Present a Heterogeneous Response to Hormonal Stimulation

**DOI:** 10.1371/journal.pone.0067697

**Published:** 2013-06-28

**Authors:** Jue Wang, Lisa Wagner-Britz, Anna Bogdanova, Sandra Ruppenthal, Kathrina Wiesen, Elisabeth Kaiser, Qinghai Tian, Elmar Krause, Ingolf Bernhardt, Peter Lipp, Stephan E. Philipp, Lars Kaestner

**Affiliations:** 1 Institute for Molecular Cell Biology and Research Centre for Molecular Imaging and Screening, Saarland University, Homburg/Saar, Germany; 2 Biophysics Laboratory, Saarland University, Saarbrücken, Germany; 3 Institute of Veterinary Physiology, Vetsuisse Faculty and the Zürich Center for Integrative Human Physiology, University of Zürich, Zürich, Switzerland; 4 Physiology, Saarland University, Homburg/Saar, Germany; 5 Experimental and Clinical Pharmacology and Toxicology, Saarland University, Homburg/Saar, Germany; University of Copenhagen and Rigshospitalet, Copenhagen, Denmark

## Abstract

Red blood cells (RBCs) are among the most intensively studied cells in natural history, elucidating numerous principles and ground-breaking knowledge in cell biology. Morphologically, RBCs are largely homogeneous, and most of the functional studies have been performed on large populations of cells, masking putative cellular variations. We studied human and mouse RBCs by live-cell video imaging, which allowed single cells to be followed over time. In particular we analysed functional responses to hormonal stimulation with lysophosphatidic acid (LPA), a signalling molecule occurring in blood plasma, with the Ca^2+^ sensor Fluo-4. Additionally, we developed an approach for analysing the Ca^2+^ responses of RBCs that allowed the quantitative characterization of single-cell signals. In RBCs, the LPA-induced Ca^2+^ influx showed substantial diversity in both kinetics and amplitude. Also the age-classification was determined for each particular RBC and consecutively analysed. While reticulocytes lack a Ca^2+^ response to LPA stimulation, old RBCs approaching clearance generated robust LPA-induced signals, which still displayed broad heterogeneity. Observing phospatidylserine exposure as an effector mechanism of intracellular Ca^2+^ revealed an even increased heterogeneity of RBC responses. The functional diversity of RBCs needs to be taken into account in future studies, which will increasingly require single-cell analysis approaches. The identified heterogeneity in RBC responses is important for the basic understanding of RBC signalling and their contribution to numerous diseases, especially with respect to Ca^2+^ influx and the associated pro-thrombotic activity.

## Introduction

Red blood cells (RBCs) display unique properties. These cells share a simple morphological structure with one single compartment, appear as single separated cells that are easily extractable [1], [2]. RBCs seem to have a high degree of uniformity but display certain variability in their hemoglobin F content, volume, shape and peripheral tissue oxygenation. However, major deviations from this normal range of variability are usually associated with pathophysiological conditions like hematuria [3], sickle cell anemia [4] or cardiovascular diseases [5].

Due to their simplicity and uniformity, RBCs have served as model systems for various processes, such as for the identification of the lipid bilayer nature of cell membranes [6]–[8] or the discovery of aquaporins [9]–[12]. Furthermore, numerous signalling molecules, signalling cascades and networks have been discovered in RBCs [13]–[16].

Beside their primary role of oxygen transport, RBC suspensions tend to aggregate under low-flow conditions or at stasis. These cells seem to play a role in thrombus formation and contribute to the development of cardiovascular diseases [17], [18]. Intercellular RBC aggregation has been shown to be evoked by exposure to lysophosphatidic acid (LPA) [19]–[21], which is released from activated platelets [22], fibroblasts, adipocytes and cancer cells [23]. LPA stimulation of RBCs is linked to a substantial increase of cytosolic Ca^2+^ [19], [24], which is readily detectable using fluorescent Ca^2+^ indicator dyes [25], [26]. Initially, LPA was thought to directly activate a non-selective cation channel in the RBC membrane [19], [26], but recent findings suggest the involvement of G-protein-coupled receptor-mediated processes [27] that are believed to be involved in numerous pathologies, such as sickle cell disease [28], hemolytic uremic syndrome [29], iron deficiency [30] and β-thalassemia [31].

Using a single-cell Ca^2+^-imaging approach, we show here that upon LPA stimulation, RBCs display individual responses differing in their kinetics and amplitude. We provide strategies for approaching the challenge of examining intercellular diversity and the analysis of such heterogeneities based on live-cell video imaging. We provide evidence that the variability of Ca^2+^ responses is at least partially related to the age of RBCs.

## Materials and Methods

### Preparation of Human and Mice Blood Samples

Experiments with human RBCs were authorized by the ethics committee of the medical association of the Saarland under registration number 132/08. Blood donors provided their written informed consent to participate in this study. This consent procedure was approved by the ethics committee of the medical association of the Saarland under the above mentioned study registration number. For the experiments, we used RBCs from healthy adult donors. Blood was drawn from a vein into heparinized syringes. Experiments with mice were carried out in strict accordance with the recommendations in the Guide for the Care and Use of Laboratory Animals of the National Institutes of Health. The protocol was approved by the State Office for Health and Consumer Protection (Permit Number: C12.4.3.4). All efforts were made to minimize suffering. Blood samples were collected from the cheeks of the mice by lancet puncture and were collected into heparinized Eppendorf tubes.

The following procedure was followed identically for RBCs from human and mice. RBCs were isolated via centrifugation at 10,000×g for 3 min. The buffy coat and plasma were discarded, and the remaining RBCs were washed three times with Tyrode solution (Tyrode) containing the following (in mM): 135 NaCl, 5.4 KCl, 10 glucose, 1 MgCl_2_, 1.8 CaCl_2_ and 10 HEPES. The pH was adjusted to 7.35 using NaOH. For imaging and flow cytometry, the cells were loaded with Fluo-4 AM (Molecular Probes, USA) at a concentration of 5 µM for 1 h at 37°C. Then, the cells were washed three times with Tyrode. All experiments were performed at room temperature (∼22°C).

### Microscopic Video Imaging to Measure Intracellular Ca^2+^

Live-cell imaging was performed to monitor intracellular Ca^2+^ kinetics in individual cells treated with LPA (Sigma-Aldrich, USA) or a biologically inactive form of LPA (1-hexanoyl-sn-glycero-3-phosphate (ammonium salt), Avanti Polar Lipids, USA). Fluo-4-loaded cells were plated onto coverslips. We waited 15 min for cell sedimentation and dye de-esterification. Fluorescence was measured on the stage of an inverted microscope (TE2000, Nikon, Japan) equipped with a 60× Plan Apo 1.4 objective. A video-imaging device (TILL Photonics, Germany) was attached to the microscope and contained a monochromator (Polychrome IV), a camera (Imago), the imaging control unit and acquisition software (TILLvision V4.0). Fluo-4-loaded cells were excited at 480 nm, and the resulting fluorescence images (using a 505 nm long pass dichroic mirror and a 535/40 bandpass filter) were collected every 5 s for 15 min. A gravity-driven local perfusion system was utilized to quickly exchange solutions in the field of view. The images were analysed in ImageJ (Wayne Rasband, National Institute of Mental Health), and the traces were further processed by IGORpro software (WaveMetrics Inc., USA) and custom-developed macros.

### Flow Cytometry to Measure Intracellular Ca^2+^

Flow cytometric measurements were performed as previously described [24]. In short, Fluo-4-loaded RBCs were analyzed using a flow cytometer (FACSCalibur, Becton Dickinson Biosciences, USA). Fluo-4 was excited at 488 nm, and the emission was collected at a centre wavelength of 530 nm. Each experiment was performed in triplicate (3 blood samples); for each measurement, 30,000 RBCs were analysed. The data were processed using BD Cell Quest Pro Software (Becton Dickinson Biosciences, USA).

### Measurement of Unidirectional Ca^2+^ Fluxes by ^45^Ca^2+^

After washing (see above), the RBCs were resuspended to a haematocrit of 10% in the incubation medium containing the following (in mM): 145 NaCl, 4 KCl, 1 CaCl_2_, 10 glucose, 10 sucrose and 10 mM Tris-HCl (pH 7.4 with NaOH). Flux measurements were initiated by the addition of ^45^CaCl_2_ (17 kBq/mL, Perkin Elmer, USA) in presence or absence of 5 µM LPA (Sigma Aldrich, USA). Samples (0.4 mL) taken immediately after tracer administration (time 0, nonspecific binding assessment) or 15 min later were washed twice with 10 mL of washing solution containing 145 mM NaCl, 4 mM KCl, 0.1 mM EGTA, and 10 TRIS-HCl buffer (pH 7.4). After washing, the cells were mixed with scintillation fluid (Quicksafe A, Zinsser Analytic). Accumulation of ^45^Ca^2+^ in the RBCs was measured using a TRI-CARB liquid scintillation analyser (Packard, Canada).

### Staining of the Reticulocytes

Staining of the reticulocytes was performed with new methylene blue (Reticulocyte stain, Sigma-Aldrich, USA) according to the manufacturer’s instruction (Figure S1). To identify reticulocytes after the video imaging experiments, the cells were stained on coverslips for 15 min at room temperature. After a gentle wash with Tyrode, the cells were air dried for 30 min and analysed. To determine the fraction of reticulocytes in mouse blood, a conventional wedge smear composed of three drops of blood and two drops of Reticulocyte stain was incubated for 10 min at room temperature, air dried for 10 min and examined with the microscope.

### Induction of Reticulocytosis in Mice

RBC populations with a high fraction of reticulocytes were prepared from BALB/c mice by repetitive bleeding according to the protocol previously described [32] with slight modifications. On day 1, the mice were anesthetized with a mixture of Xylazine at 17.5 mg per kg body weight (Rompun, Bayer, Germany) and Ketamine at 85 mg per kg body weight (Ursotamin, Serumwerk Bernburg, Germany). The animals were then treated with an intraperitoneal injection of 2 mL 0.9% NaCl solution, and 500 µL of blood was drawn by retro-orbital puncture. The procedure was repeated on day 3. During this period, it was essential to supplement the drinking water with iron (150 µg/mL FeSO_4_ **⋅**7H_2_O) and folate (0.2 µg/mL) to compensate for iron loss**.**

### PKH26-staining of Mouse RBCs

After induction of the reticulocytosis, 500 µL of blood was drawn by retro-orbital puncture on day 5. Plasma was separated by centrifugation and discarded. The RBCs were stained with PKH26 (Sigma, Germany) according to the manufacturer’s protocol: 250 µL of RBCs were mixed with 2.25 mL diluent C from the PKH26 cell linker kit and then incubated with 2 µM PKH26 dye for 5 min at room temperature. The staining reaction was stopped by the addition of 2.5 mL 1% bovine serum albumin for 1 min, followed by dilution of the BSA with 5 mL 0.9% NaCl solution. The cells were washed three times with 0.9% NaCl solution at 400×g for 10 min at room temperature, resuspended in a final volume of less than 300 µL and injected back into the mice.

### Analysis of PKH26-stained Cells

Forty-three days after the re-injection, blood samples were collected from the cheeks of the mice. After this period, PKH26 positive cells were referred to as ”old” cells and were isolated by fluorescence-activated cell sorting (FACS) using a Modular Flow (MoFlo) cell sorter (Beckman Coulter, USA). For microscopic identification of PKH26-positive cells, a TRITC filter-set was used (excitation: 550 nm; emission: 575–630 nm). For information on the removal of PKH26 crosstalk in the Fluo-4 recording channel, refer to Figure S2.

### Enzymatic Assays

PKH26 positive (old cells) and PKH26 negative cells were isolated as described above. To discriminate reticulocytes, RBCs obtained after retro-orbital puncture were stained with phosphatidylethanolamine labeled anti-CD71 antibody (Southern Biotech, USA). CD71 positive and CD71 negative cells were separated by flow cytometric cell sorting using a FACSAria III (BD Biosciences, USA).

Acetylcholinesterase (AChE) activity of 2×10^6^ RBCs of each population were performed using a colorimetric AChE assay kit (Amplite, AAT Bioquest, USA) following the manufacturers instructions. Measurements were performed in a detection volume of 5 µL using a fibre-optic ultra-micro cell (TrayCell, Hellma Analytics, Germany) and an absorption spectrometer (Lambda Bio+, Perkin Elmer, USA).

To test the pyruvate kinase activity in reticulocytes, 10^7^ CD71 positive cells were exposed to 5 µM LPA or incubated in Tyrode solution for 15 min and subsequently shock frozen in liquid nitrogen. Pyruvate kinase activity was determined using a kit (Cayman Chemical Company, USA) following the manufacturers instructions and an Infinite M200 fluorescence plate reader (Tecan, Switzerland).

### Confocal Measurements

Confocal recordings were performed on a Leica TCS SP5 microscope (Leica, Germany) as described recently [33]. Fluorescence images were acquired as follows: excitation at 488 nm, emission 491–540 nm for Fluo-4; excitation at 561 nm, emission 565–630 for phosphatidylethanolamine labelled CD71 or PKH26; and excitation at 633 nm, emission 638–750 nm for allophycocyanin (APC) labelled annexin V (Medsystems, UK). Successive recordings of each fluorescence channel allowed crosstalk free imaging.

### Data Analysis

Hill’s equation was used for fitting F/F_0_ traces of single cells:
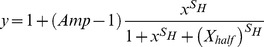
where *Amp* was the maximal value of the fitted curve, referred to as amplitude in the paper; *S_H_* was the Hill slope of the curve, and *X_half_* was the x value at 50% of the amplitude. Based on this equation, a macro was designed in IGORpro to allow for an automated analysis of the fluorescence intensity curves. Analysis was performed either on the entire population of cells or on the subpopulation of responding cells. A cell was referred to as a responder when its fluorescence intensity increase exceeded 3 times the standard deviation of the fluorescence recorded during control conditions. All data are presented as the mean values ±SEM of three replicate experiments unless otherwise stated. The comparison between the experimental groups was performed using a normality test followed by a two-tailed Student's t-test for Gaussian distributed values of unpaired samples (GraphPad Prism 4, USA). Non-Gaussian distributed values were tested for significant differences with a Mann-Whitney test. The level of statistical significance was indicated as p<0.05 (*), p<0.01 (**) or p<0.001 (***).

## Results

### Properties of Lysophosphatidic Acid-induced Ca^2+^ Influx

**Figure 1:**
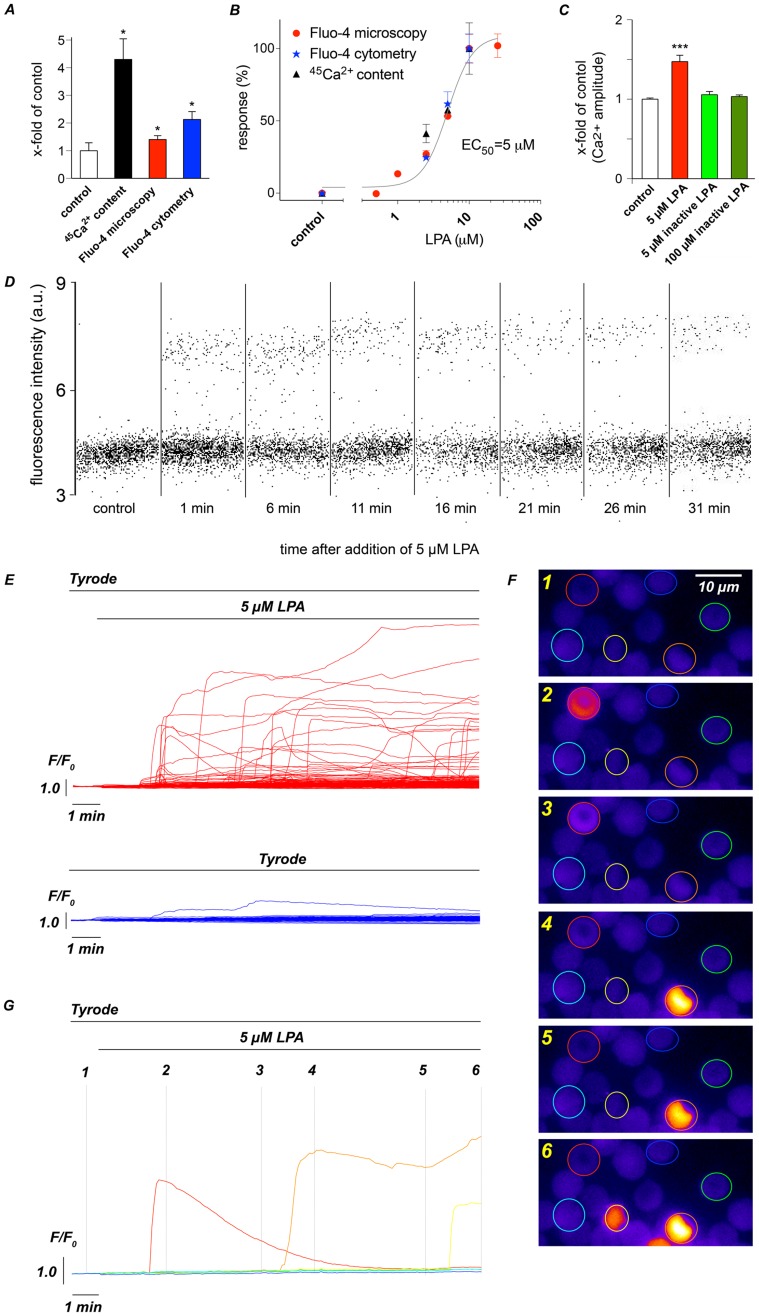
Ca^2+^ increase in RBCs stimulated by the LPA: Methodological approaches and variable responses. (A) Comparison of Ca^2+^ increase after 15 min of 5 µM LPA treatment compared to a normalized response under control conditions for radioactive Ca^2+^ as a tracer, Fluo-4-based video imaging and Fluo-4-based flow cytometry. To avoid interindividual differences (see Figure S3), all measurements were performed with freshly prepared samples from a single healthy donor. All measurements showed a significant increase in Ca^2+^ compared to control conditions. The error bar on the control-column refers to the ^45^Ca^2+^-measurements. Error bars for video imaging and flow cytometry control values are smaller and have been omitted for reasons of lucidity. (B) Normalized dose-response behaviour measured by the methodologies mentioned in (A) revealing an EC_50_ for LPA of 5 µM. The values for Fluo-4 microscopy are taken from Kaestner *et al.* 2012 [47]. (C) Ca^2+^ increase measured by video imaging after application of 5 µM LPA or a biologically inactive form of LPA (06∶0 Lyso PA) at a concentration of 5 and 100 µM. (D) Flow cytometry data at different time points after starting LPA treatment. (E) Changes in the intracellular Ca^2+^ levels in single cells visualized by fluorescence imaging of the Ca^2+^ sensor Fluo-4 in response to 5 µM LPA (upper red traces) and under control conditions (lower blue traces). The duration of LPA exposure is indicated at the top of the traces. (F) Images of Ca^2+^ signals of a representative group of cells (labelled by coloured circles) at different time points. (G) LPA response of selected cells shown in (F) (colour matched) with time points of each image as indicated.

Lysophosphatidic acid (LPA)-induced Ca^2+^ influx into RBCs can be measured by several methodologies, including radioactive measurements of unidirectional tracer fluxes utilizing ^45^Ca^2+^ or by fluorescence-based techniques, such as flow cytometry and microscopic imaging. The first two methods have been well established for several decades [34]–[37], whereas live-cell imaging of RBCs for Ca^2+^ was introduced just a couple of years ago [25], [26] and has been used scarcely ever since [38]. To compare the methods, human RBCs from a single donor were stimulated with 5 µM LPA for 15 min, and the signals obtained were normalized to controls treated with Tyrode (Figure 1A). All of the approaches demonstrated a significant increase in intracellular Ca^2+^ following LPA stimulation but albeit to a different degree of change.

Measurements of ^45^Ca^2+^ revealed a substantially higher signal compared to fluorescence-based techniques, most likely because the total amount of Ca^2+^ entering the cell was detected, whether it was bound to proteins or soluble. In contrast, the fluorescence-based approaches exclusively detect free Ca^2+^. Thus, our data are in agreement with previous investigations reporting that approximately 80% of intracellular Ca^2+^ in RBCs is not free but is bound to intracellular constituents [39], [40]. Fluo-4-based microscopy did not show significant differences compared to flow cytometry (p = 0.57).

To confirm these findings, we analysed Ca^2+^ signals in RBCs at different LPA concentrations and calculated the relative dose-response relationships. As depicted in Figure 1B, the dose-response curves for all three approaches were superimposable, indicating that Ca^2+^ signals of RBCs can be reliably measured with all three methods.

However, ^45^Ca^2+^ flux measurements do not allow for the analysis of individual RBCs, whereas the other two techniques reveal single-cell responses. To exclude unspecific lipid interactions, we performed Ca^2+^-imaging experiments with an inactive form of LPA (06∶0 Lyso PA, Figure 1C) revealing no difference to control conditions even at concentrations of 100 µM.

Figure 1D depicts a flow cytometric analysis showing that the RBCs responded variably. Only a few (17%) of the cells showed an intracellular Ca^2+^ increase in response to LPA stimulation.

In contrast, single-cell live imaging provides Ca^2+^ signals with high spatio-temporal resolution. As shown in Figure 1E–G, the method revealed that LPA-induced Ca^2+^ responses appear rather heterogeneous between individual human RBCs. Such a degree of heterogeneity is reported here for the first time and is a rather unexpected finding that contrasts the scientific consensus of RBCs as a homogenous population of cells with very similar properties. The variable responses of RBCs were not restricted to human donors, as we found similar variability in the RBCs from mice (Figure S4).

### Protocols and Parameters to Characterize and Analyse LPA-induced Ca^2+^ Influx

**Figure 2:**
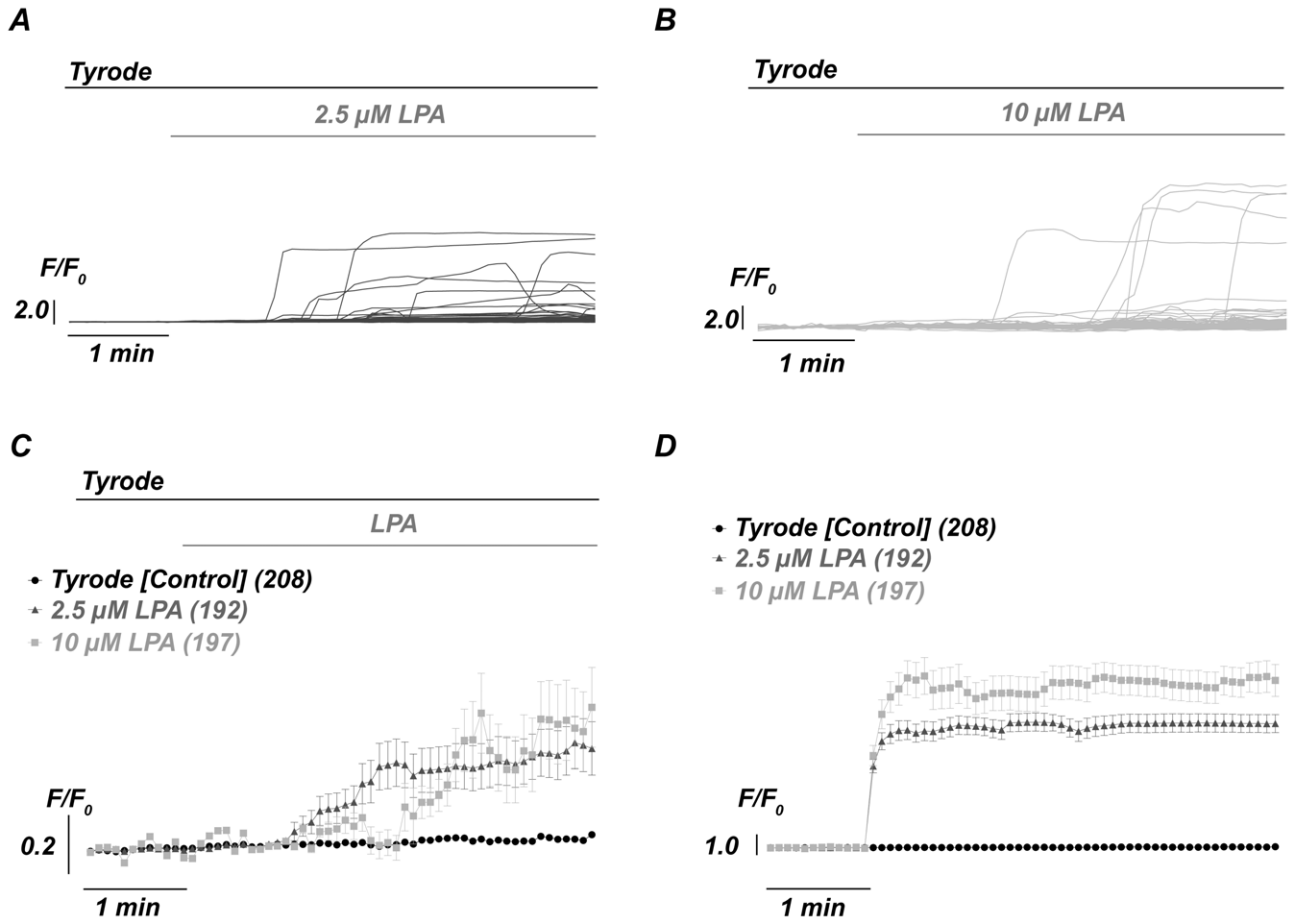
Handling and protocols of LPA-induced Ca^2+^ influx. Fluorescence signals of single RBCs treated with 2.5 µM LPA (A) and 10 µM LPA (B). (C) Comparison of average Ca^2+^ signals induced by different concentrations of LPA [same data as in (A) and (B)]. (D) Average of the Ca^2+^ signals after their synchronization to the onset of the response.

Live-cell video imaging allows for the analysis of individual cells and therefore appeared to be most appropriate to determine intracellular Ca^2+^ changes when compared to other techniques considered here (Figure 1A, B). In light of the complexity of single-cell responses (Figure 1E), we wanted to determine whether a simple averaging of all cells might be an adequate approach. To evaluate the best method of analysis, we compared Ca^2+^ signals under control conditions and in the presence of 2.5 and 10 µM LPA (Figure 2A, B). Figure 2C shows the averaged traces of the cellular responses. The traces appear shallow, small and independent of the LPA concentration. However, this finding might be a misinterpretation because the onsets of the responses of each single cell differed largely (Figure 2A, B). Therefore, we synchronized the responses to their onset before averaging. As demonstrated in Figure 2D, the RBCs generated steep Ca^2+^ increases with an amplitude that was dependent on the LPA concentration. Thus, post-experimental synchronization might be adequate to analyse LPA-induced Ca^2+^ response in RBCs. It is noteworthy to mention that a full characterization of the RBC responses to external stimulation might be associated with a long-lasting delay period. Therefore, the recording time (limited here to 15 min) should be extended to obtain a maximum of responding cells. However, an extension of these experiments is limited because RBCs with high intracellular Ca^2+^ levels tend to have a fragile plasma membrane with an increased risk of membrane breakdown.

**Figure 3:**
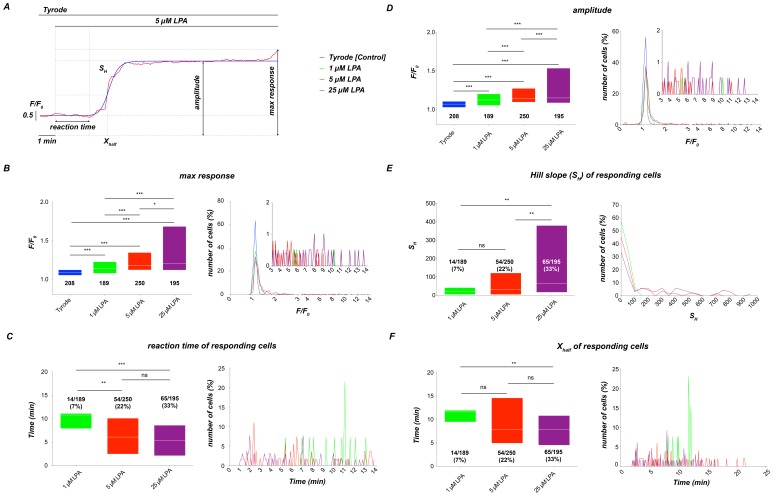
Different parameters of single-cell response. (A) Definition of the different parameters related to the cellular response. (B)–(F) Statistical analysis of the parameters defined in (A); the colour-code for all diagrams is given in the right part of the panel. To avoid interindividual differences (see Figure S3), all measurements were performed with freshly prepared samples from a single healthy donor. (B) Maximal intensity of the cellular response within the period of measurement (max response). (C) Time interval between LPA application and the onset of the reaction (reaction time). (D) Value of the main plateau or the major peak of the Ca^2+^ response (amplitude). (E) Hill slope (steepness) of the Ca^2+^ increase (S_H_). (F) The time point when the ratio (F/F_o_) reached the value of half the amplitude (X_half_). The values in (D)–(F) are extracted from a Hill-equation fitting. Parameters in (B) and (D) are derived from the total number of cells, while (C), (E) and (F) refer exclusively to responding cells and therefore do not give a number for the control condition. The numbers below the boxes give the cell numbers taken from three blood samples.

Therefore, we also considered achieving cellular synchronization using a three-step protocol (see Figure S5). However, this approach required blocking the plasma membrane Ca^2+^-pump with sodium orthovanadate, which complicates the interpretation and comparison with other protocols. Such a multi-step protocol might be appropriate if the pure LPA mediated influx capacity but not the physiological response of the cells is in question. Furthermore, kinetic information on the Ca^2+^ signals would be lost in such a multi-step protocol. Consequently, we focused on the stimulation protocol shown in Figure 2, and examined a large number of parameters from individual responses, as depicted in Figure 3A. Each single-cell fluorescence trace was fitted with a Hill equation as outlined in the Materials and Methods section, which allowed us to extract numerous types of quantitative information, such as the cellular reaction time (time between LPA application and the onset of the Ca^2+^ signal), the time for half-maximal stimulation (“X_half_”), the steepness of the upstroke (Hill slope or “S_H_”) and the amplitude of the cellular response (“amplitude”).

Such parameters were analysed statistically as depicted in Figure 3B–F (left) for an experimental series in which we tested the effects of varying LPA concentrations. We found significant differences in all parameters, indicating that the LPA concentration significantly impacted all the parameters. It is noteworthy that the distribution of the analysed parameters for responding cells (see histograms and their insets in Figure 3B–F) was characterized by a broad scattering rather than a specific distribution. Therefore, we investigated the putative reasons underlying the scattered distribution of the cellular responses.

### The Influence of Cell Age on LPA-induced Ca^2+^ Influx and Ca^2+^ Effectors

Cellular properties change with cell age, which also applies to RBCs [41], [42]. An established method to differentiate RBCs by age is a separation using Stractan or Percoll density gradients. This approach is based on the assumption that RBCs gain density with age. However, recent findings indicated that the densification with age reversed in cells approaching the age of clearance [43] and that those methods may activate receptors or channels in the RBCs′ plasma membranes [[44] and unpublished results of the Bogdanova lab]. This change might lead to a dehydration of young cells and their movement to the fraction of dense cells. Although such effects may only apply to a subpopulation of the separated fractions, we used a more reliable and reproducible approach to compare LPA-induced Ca^2+^ signals of very young RBCs (reticulocytes) and RBCs on the verge of clearance (i.e., very old). Because this approach was not applicable to RBCs of human donors, we used the RBCs of BALB/c mice for the remainder of this report.

#### LPA-induced Ca^2+^ influx and Ca^2+^ effectors in reticulocytes

**Figure 4:**
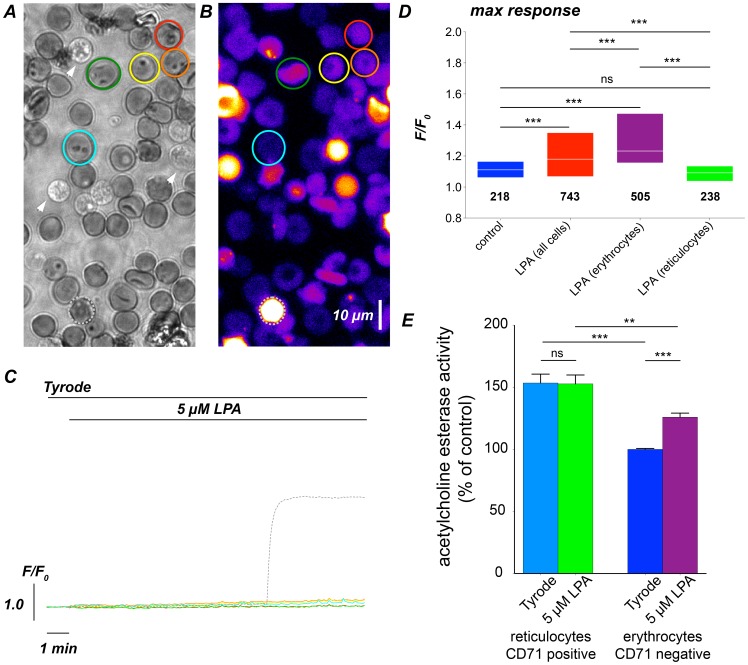
Ca^2+^ response of reticulocytes to LPA stimulation. (A) A representative image of a new methylene blue staining of RBCs from a BALB/c mouse after induction of reticulocytosis. The coloured regions depict reticulocytes analysed in (B) and (C). The arrowheads point to lysed RBCs. (B) Image of Fluo-4 loaded live RBCs. The coloured regions are transferred from (A). The dashed grey circle labels a responding RBC. (C) Intensity traces for Ca^2+^ content of the cells marked in (B) stimulated with LPA. (D) Statistics of the maximal response of reticulocytes and the entire RBCs population without reticulocytes (referred to as erythrocytes) under control conditions and for 5 µM LPA stimulation. The numbers below the boxes give the cell numbers taken from three mice. (E) AChE activity in reticulocytes and erythrocytes with and without stimulation with 5 µM LPA for 15 min. The measurements comprise of a colorimetric assay based on 2×10^6^ cells per measurement and the data is the average of 5 mice.

We identified reticulocytes following the Ca^2+^-imaging experiments by new methylene blue staining (Figure 4A). Because the normal fraction of reticulocytes in the blood is very low (approximately 1%), we increased their number by reticulocytosis [32]. As depicted in the right panel of Figure S1A, the substantial increase in the number of reticulocytes was apparent following this intervention. We then challenged different cell populations with 5 µM LPA and analysed their Ca^2+^ response (Figure 4B–D). Such, we found that in comparison to the fraction of erythrocytes (purple box in Figure 4D), the reticulocytes (green box in Figure 4D) did not show any response to the LPA stimulation.

Increased acetylcholinesterase (AChE) activity is regarded as a marker of RBC membrane alteration and RBC aging [45], [46]. Therefore we tested AChE activity in reticulocytes and older RBCs (erythrocytes). To do so, reticulocytes were stained with anti-CD71 antibody and separated by FACS. Cells of both populations were treated for 15 min with 5 µM LPA and their AChE activity was compared to unstimulated cells. The results are depicted in Figure 4E. As expected the AChE activity of CD71 negative cells was largely reduced compared to CD71 positive reticulocytes. However, in contrast to CD71 negative cells reticulocytes did not respond to LPA stimulation with an increase in of AChE activity indicating that their membrane integrity remained unaffected by LPA.

**Figure 5:**
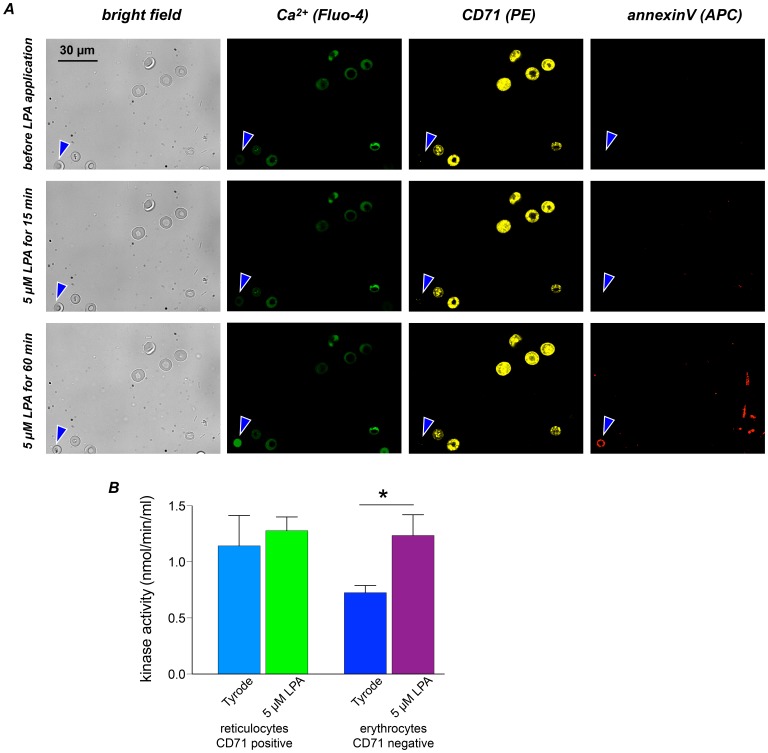
LPA-induced Ca^2+^ effectors in reticulocytes. (A) Bright field and confocal images of reticulocytes just before, 15 min after and 60 min after stimulation with 5 µM LPA. The white light images in the left column provide an overview. The blue arrowhead marks a CD71 negative erythrocyte. The cell clearly displays a Ca^2+^ signal (visualised in green) as well as annexin V staining (visualised in red) indicating PS exposure. For the CD71 positive stained reticulocytes the intracellular Ca^2+^ concentration remained at low levels and an annexin V staining is absent. (B) Pyruvate kinase assay of sorted CD 71 positive and CD71 negative RBCs. Reticulocytes (CD71(+)) displayed no significant change of kinase activity upon stimulation with 5 µM LPA. CD71 negative RBCs showed similar activity after LPA stimulation but their steady state activity before stimulation was significantly reduced. Each bar is the mean of measurements of cells from four mice.

Next we analysed Ca^2+^ effector mechanisms in reticulocytes. Phosphatidylserine (PS) translocation from the inner to the outer membrane leaflet is known to be triggered by Ca^2+^ [47]–[49] and was therefore analysed at the same time as the intracellular free Ca^2+^ concentration following LPA stimulation (Figure 5A). Confocal images confirmed the absence of Ca^2+^ signals in reticulocytes within 60 min after 5 µM LPA stimulation and revealed the absence of PS at the cell surface in the same period. Furthermore we analysed pyruvate kinase activity, which has been reported to be Ca^2+^-dependent [50]. The data presented in Figure 5B demonstrate a significant increase of kinase activity after 5 µM LPA stimulation in CD71 negative erythrocytes, but no increase in reticulocytes, whatsoever.

#### LPA-induced Ca^2+^ influx and Ca^2+^ effectors in old RBCs

**Figure 6:**
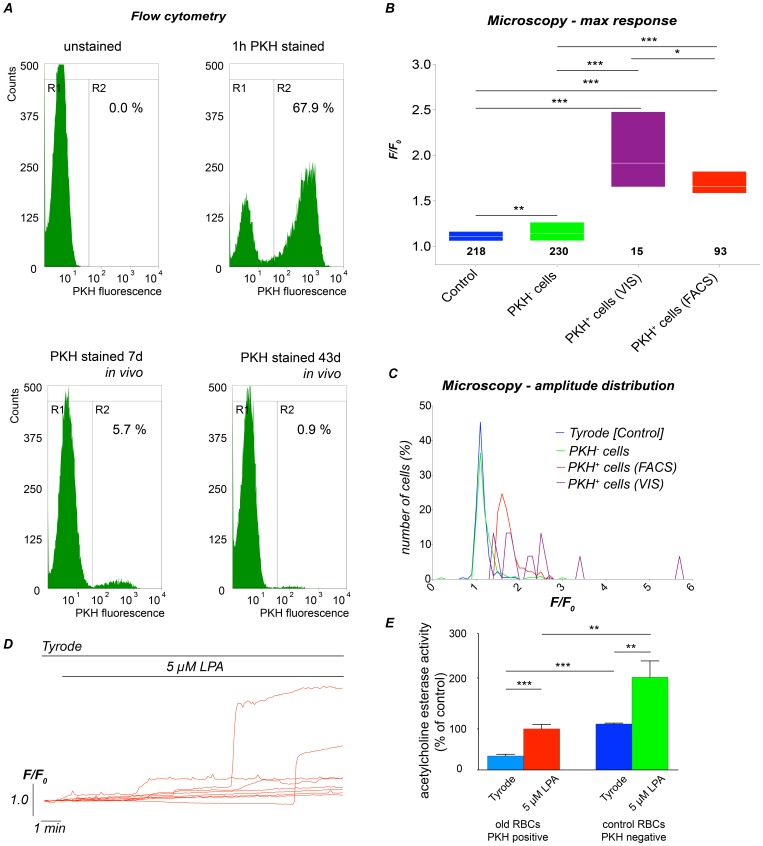
Ca^2+^ response of old RBCs to LPA stimulation. (A) Analysis of PKH26 fluorescence of 20,000 RBCs by flow cytometry before (upper left) and 1 hour after staining with PKH26 (upper right). After the staining procedure, the cells were reinjected into the same mice and analysed 7 days (lower left) and 43 days (lower right) later. The percentage of PKH26 labelled cells (PKH(+), region R2) is indicated. (B) Control and LPA stimulation experiments were performed on PKH26-positive (+) and PKH26-negative (–) RBCs. The maximal response under the different conditions is given. We discriminated between PKH(+) cells identified directly under the microscope (VIS, low in number) and RBCs sorted by FACS. The numbers below the boxes give the cell numbers taken from three mice. (C) Amplitude histogram of the RBC treated under the conditions mentioned in (B). (D) Representative intensity traces of PKH(+) cells stimulated with 5 µM LPA revealing a high heterogeneity also in old RBCs. (E) AChE activity in control and old RBCs with and without stimulation with 5 µM LPA for 15 min. The measurements comprise of a colorimetric assay based on 2×10^6^ cells per measurement and the data is the average of three mice.

To identify very old RBCs at the verge of clearance, we drew blood from mice subjected to reticulocytosis and stained RBCs with the plasma membrane stain PKH26 [51] (Figure 6A, upper panels). At least 67.9% of the cells that were re-injected into the same mouse were stained with PKH26. The fluorescence of the cells was analysed again after 7 and 43 days (Figure 6A, lower panels). After 7 days in circulation, 5.7% of the cells were PKH26-stained; after 43 days, this portion was reduced to less than 1%, indicating that the rest of the PKH26 positive erythrocytes were cleared in the mouse body. Because the average lifetime of RBCs in BALB/c mice has been determined to be 46 days [52], we waited for 43 days until we isolated PKH26-stained RBCs by fluorescence activated cell sorting (region R2 in Figure 6A). Those cells were regarded as old cells close to clearance. Ca^2+^ signals were compared to non-stained RBCs representing cells of all ages. Figures 6B–D summarize the results obtained with these two cell populations. PKH26-negative cells responded only with a very small but significant increase when stimulated with 5 µM LPA (green box in Figure 6B). In contrast, the PKH26-positive cells that were manually selected from the original blood sample (purple box in Figure 6B) and the PKH26-positive cells enriched by FACS (red box in Figure 6B) both displayed a substantially augmented Ca^2+^ response. Nevertheless, neither visually identified PKH^+^ cells (VIS) nor FACS sorted cells showed LPA responses that were uniform. Instead, both population of responders still displayed a substantial heterogeneity, as observed in the response histograms in Figure 6C and in the representative traces in Figure 6D.

In line with previous data [46] we found that the AChE activity in very old cells was largely reduced to 30% of control cells (Figure 6E). In contrast to reticulocytes (Figure 4E) old RBCs still responded to LPA but their AChE activity did not exceed the values of unstimulated control cells.

**Figure 7:**
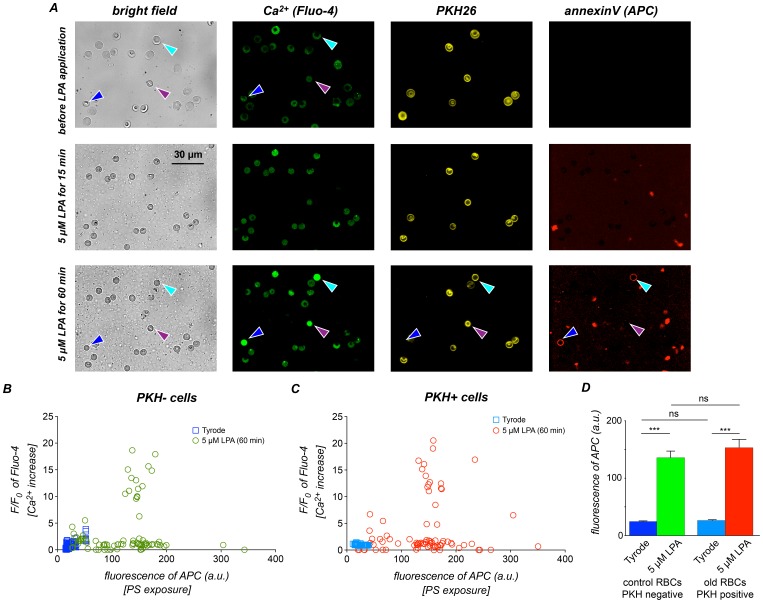
Phospatidylserine exposure of old RBCs after LPA stimulation. (A) Bright field and confocal images of reticulocytes just before, 15 min after and 60 min after stimulation with 5 µM LPA. The white light images in the left column provide an overview. The blue arrowhead marks a “control” RBC, because the PKH26 staining (visualised in yellow) is missing. The cell clearly shows a Ca^2+^ signal (visualised in green) as well as an annexin V staining (visualised in red) indicating PS exposure. The cells marked with cyan and purple arrowheads represent the diversity of the old (PKH26 positive) cells. Although both cells display an elevated intracellular Ca^2+^, they differ in their PS exposure, which is present for the cyan marked RBC and absent for the purple marked. (B) Analysis of microscopy images of PKH26 negative RBCs without and after 60 min of 5 µM LPA stimulation concerning the relation between increased intracellular free Ca^2+^ and PS exposure. (C) the same as (B) but for PKH26 positive cells. (D) average PS exposure in control and old RBCs without and after 60 min of 5 µM LPA stimulation. Cells in (B) to (D) derive from four mice.

We next analysed PS exposure in old RBCs after LPA stimulation by confocal microscopy (Figure 7). Singe cell analysis (Figure 7A) revealed that the PS exposure adds another level of heterogeneity to the hormonal response of RBCs: Cells with similar Ca^2+^ signals showed different amounts of PS exposure (cyan and purple arrowheads in Figure 7A). Furthermore, even cells without a Ca^2+^ signal displayed an increased PS exposure (Figure 7B, C). This observation, however, was independent of the cell age in terms of very old cells vs. an average RBC population as there is no difference between PKH26 positive and negative cells (Figure 7D).

In summary, the age of RBCs appeared to be an important factor responsible for the heterogeneity of LPA-induced responses. However, our data also indicate that the age of RBCs is not the only characteristic responsible for the observed variability.

## Discussion

Here, we show that although RBCs have a high morphological homogeneity, they are functionally heterogeneous (Figure 1). Despite extensive past investigations of RBCs (they are among the most studied cells in natural history), this heterogeneity was widely ignored because bulk or suspension experiments could not reveal such properties. Nevertheless, other single-cell experiments, namely the patch-clamp technique, may have led to similar conclusions due to discrepancies between different laboratories, e.g., [53] vs. [54] and [55] vs. [56] or heterogeneous results within a laboratory [57]–[59] where standardized conditions were applied. However, the inherent complexity of the technique combined with relatively small cell numbers rendered conclusions difficult [60].

To exclude putative artefacts of the LPA stimulation experiments we followed three strategies: (i) the Ca^2+^ increase was measured by three independent techniques (Figure 1A, B), (ii) experiments with an biologically inactive form of LPA, namely 06∶0 Lyso PA, were performed (Figure 1C) and (iii) plasma membrane integrity of the RBCs was probed by an AChE activity assay (Figures 4E and 5E). The latter one revealed a decrease of AChE activity with RBC age that is in agreement with previous investigations [46], [61]. The facts that (i) LPA stimulation did not change AChE activity in reticulocytes (Figure 4E) and (ii) AChE activity in LPA stimulated old RBCs did not exceed AChE activity in control RBCs at rest (Figure 5E), allows in combination with the 06∶0 Lyso PA experiments (Figure 1C) the conclusion that 5 µM LPA stimulation does not alter RBC membrane integrity. Modulations of AChE activity upon LPA stimulations are more likely the result of the increase in intracellular free Ca^2+^ [62] than disturbances in membrane structure.

A major result is that we provided novel approaches to quantitatively analyse RBC responses to processes such as hormonal stimulation on the level of individual cells (Figure 3) that are not accessible by any other technique.

Heterogenic Ca^2+^ responses from RBCs include a variable delay (reaction time) of the Ca^2+^ entry, a common biphasic increase, differences in amplitudes and an occasional decrease of Ca^2+^ after the peak (Figures 1E, 2A, 2B, 5D and Figure S4A).

Cell-age, the exclusive known source of any heterogeneity to date, contributes to the intercellular differences but cannot fully explain them (Figures 4–7). However, we could identify the following stereotypic aspects in responses from the reticulocytes and old RBCs: (i) reticulocytes did not respond to LPA stimulation at all, neither in terms of Ca^2+^ entry, AChE activity, PS exposure nor pyruvate kinase activity, and (ii) almost all RBCs close to the end of their life cycle responded to LPA stimulation with a Ca^2+^ entry (Figure 6C); the response kinetics was still heterogeneous (Figure 6D), and the median amplitude was consistently higher than the one from a unseparated population of mixed age (Figure 6B). This finding can partly be attributed to a decrease in the Ca^2+^-pump activity with increasing cell age [43]. Additionally, a Ca^2+^ effector mechanism, the PS exposure to the outer membrane leaflet presents an increased degree of variability, which in old RBCs is similar compared to control cells (Figure 7).

The molecular source of the variable behaviour in general is still unclear and needs further investigation. The nature of RBCs excludes regulation of protein expression. Potential regulation mechanisms include protein translocations, post-translational modifications or degradations. Currently, the test of such mechanisms remains difficult because little is known about the signal transmission between putative LPA receptor activation and the means of Ca^2+^ entry, which could be new or known transport proteins [63]–[65].

In general, our finding of different and variable Ca^2+^ signals and PS translocations in the morphologically homogeneous population of RBCs appears important for our understanding of RBC physiology and pathophysiology. This finding especially holds true because Ca^2+^ entry in RBCs is not solely initiated by LPA stimulation, but has also been reported for other hormonal and lipid based stimulations, such as with arachidonic acid [66], prostaglandin E_2_ [25] or homocysteine [67]. In RBCs, many different effector mechanisms are directly or indirectly influenced by the intracellular Ca^2+^ concentration, such as the activation of (i) the Gardos channel, (ii) calpain cleavage, (iii) conventional protein kinases, (iv) the scramblase, (v) the plasma membrane Ca^2+^-pump and (vi) the inhibition of the flippase [38], [40]. These Ca^2+^ induced molecular mechanisms are associated with the cellular death of RBCs [15]. Surprisingly, the PS translocation to the membrane surface, which is believed to be mediated by the Ca^2+^ dependent scramblase [48], [68], shows an even higher heterogeneity than the Ca^2+^ signal, but it was independent of the RBC age in terms of very old RBCs vs. an average mixed population.

Furthermore, there is emerging evidence that RBCs might even play an active role in the process of thrombus formation, and Ca^2+^ appears to be central for this process as well [17], [21], [69]. In this way, understanding heterogeneous Ca^2+^ signalling might be important to understand processes like local thrombus formation. Ca^2+^ signalling may transform the RBC into an active contributor to thrombosis; even if the contribution is small, it might be globally or locally relevant when occurring on top of pre-existing pathologies. Furthermore, there is growing clinical evidence in support of Ca^2+^-associated pro-thrombotic activity of RBCs in diseases such as β-thalassemia [31], [70], sickle cell disease [71], [72] or malaria [73], [74].

Nevertheless, it is noteworthy that not all aspects of Ca^2+^ influx are negative; a small intracellular Ca^2+^ increase to 200 nM can also be beneficial due to a higher O_2_-binding affinity (Makhro et al., N-methyl D-aspartate receptors in erythroid precursor cells and in circulating human red blood cells contributes to the regulation of intracellular calcium levels, in revision for publication). Therefore, the hormonal stimulation could also tune physiological performance, and under certain conditions it might even govern the local control of hormone release.

In conclusion Ca^2+^ signalling in RBCs is surprisingly dynamic and diverse. RBC age is just one of the determinants for the diversity. These unexpected findings need to be taken into account in further studies that require approaches for single-cell analysis. The identified heterogeneity in the RBC response described in our study is important because it not only impacts our basic understanding of RBCs signalling but also our understanding of their contribution to numerous diseases. Taken together with the emerging knowledge of an active role of RBCs in blood clotting, understanding the dynamics of RBC Ca^2+^ signalling might offer new targets for modulating thrombotic activity.

## Supporting Information

Figure S1 **Staining of reticulocytes with New Methylene Blue.** (A) The images depict typical samples of blood smear stains under control conditions (left image) and after induction of a reticulocytosis (right image). (B) The cells marked 1–4 [enlargements of the cells marked with arrows in (A)] show the developmental stages from reticulocytes to adult erythrocytes.
(TIF)

Figure S2 **Double-staining of mouse RBCs with Fluo-4 and PKH26.** (A) Fluo-4-loaded cells do not show a cross talk into the “red” recording channel. (B) PKH26-stained cell recorded in the “red channel” depict a cross talk of 15% into the “green channel”. (C) Double-stained “raw images” in the green and red recording channels. (D) Double-stained images [same as in (C)] corrected for the PKH26 crosstalk. The scale bar for all images represents 20 µm.
(TIF)

Figure S3 **Average Ca^2+^ signals after 5 µM LPA stimulation for 3 individual healthy donors indicating the degree of inter-individual variations.**
(TIF)

Figure S4 **LPA stimulation of mouse RBCs.** (A) Single-cell fluorescence response of mouse RBCs after stimulation with 5 µM LPA. (B) Dose response relationship of the LPA concentration with a calculated EC_50_ of 3.3 µM, which is close to the value for human RBCs (5.0 µM) (compare to Figure 1).
(TIF)

Figure S5 Panel (A) depicts the attempt to “synchronize” the cells by applying a three step protocol starting with the application of a Ca^2+^ free solution and inhibition of the Ca^2+^ pump with sodium orthovanadate (SOV). Then, the RBCs were stimulated with LPA for 5 min, and Ca^2+^ (1.8 mM) was added, leading to a synchronized cell response. (B) Single-cell traces show [same data as in (A)] that the cells still respond variably to the Ca^2+^ readdition.
(TIF)
