# Supplementary figures and images for: Morphologically Homogeneous Red Blood Cells Present a Heterogeneous Response to Hormonal Stimulation
Source: PLoS One. 2013 Jun 28;8(6):e67697. doi: 10.1371/journal.pone.0067697 (PMC3695909; doi:10.1371/journal.pone.0067697)

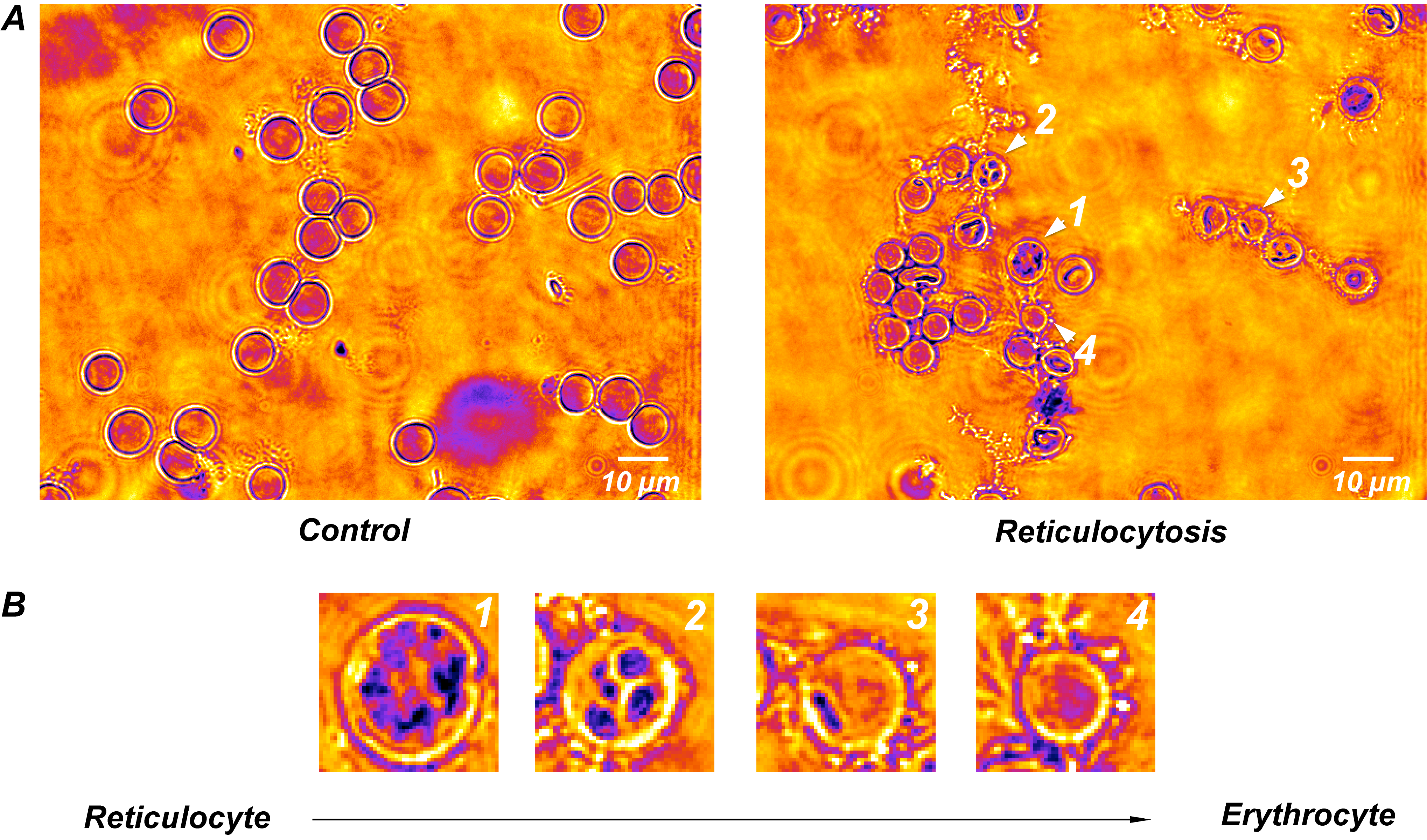

Supplement: Figure S1 — Staining of reticulocytes with New Methylene Blue. (A) The images depict typical samples of blood smear stains under control conditions (left image) and after induction of a reticulocytosis (right image). (B) The cells marked 1–4 [enlargements of the cells marked with arrows in (A)] show the developmental stages from reticulocytes to adult erythrocytes. (TIF) [file pone.0067697.s001.tif]

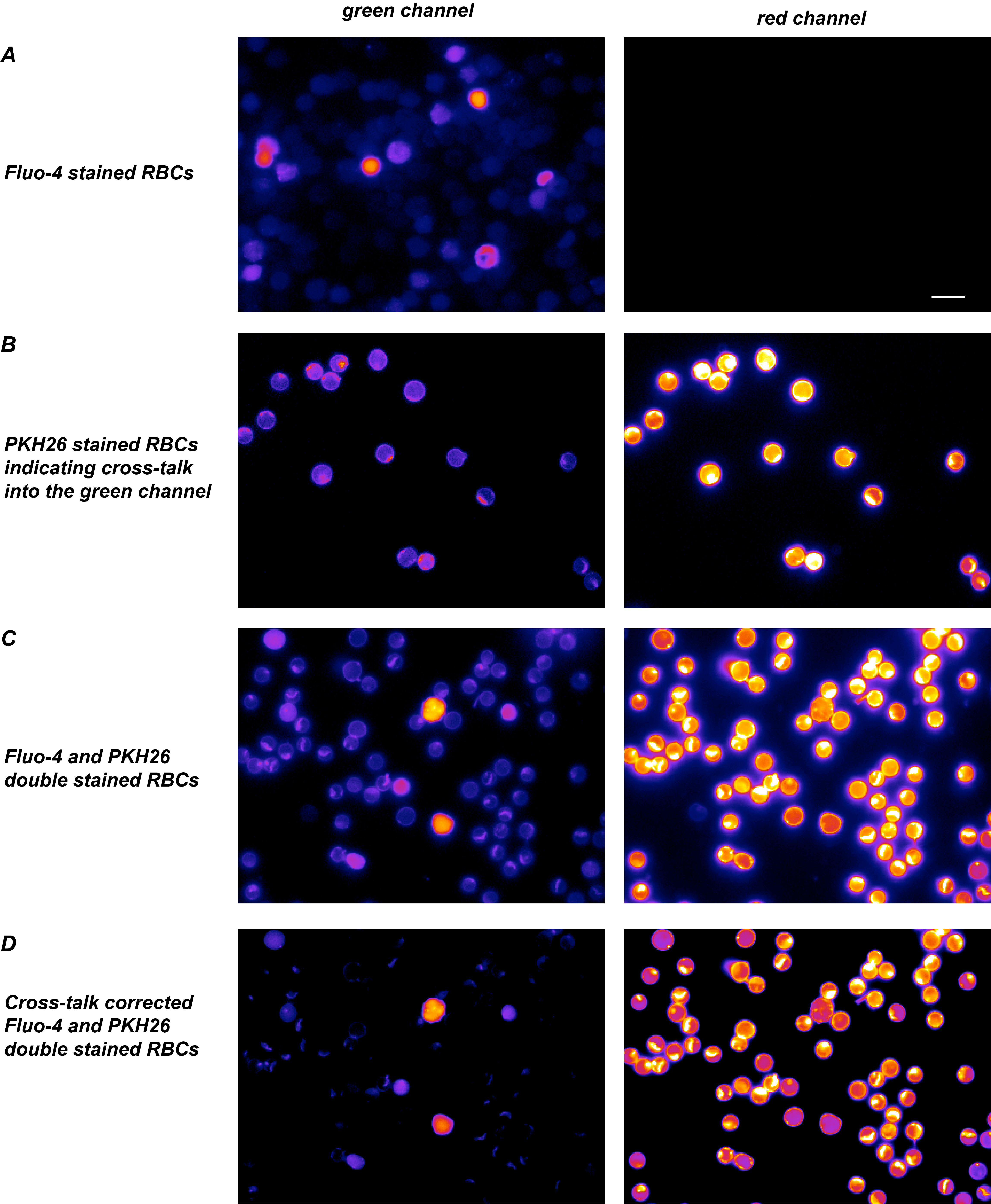

Supplement: Figure S2 — Double-staining of mouse RBCs with Fluo-4 and PKH26. (A) Fluo-4-loaded cells do not show a cross talk into the “red” recording channel. (B) PKH26-stained cell recorded in the “red channel” depict a cross talk of 15% into the “green channel”. (C) Double-stained “raw images” in the green and red recording channels. (D) Double-stained images [same as in (C)] corrected for the PKH26 crosstalk. The scale bar for all images represents 20 µm. (TIF) [file pone.0067697.s002.tif]

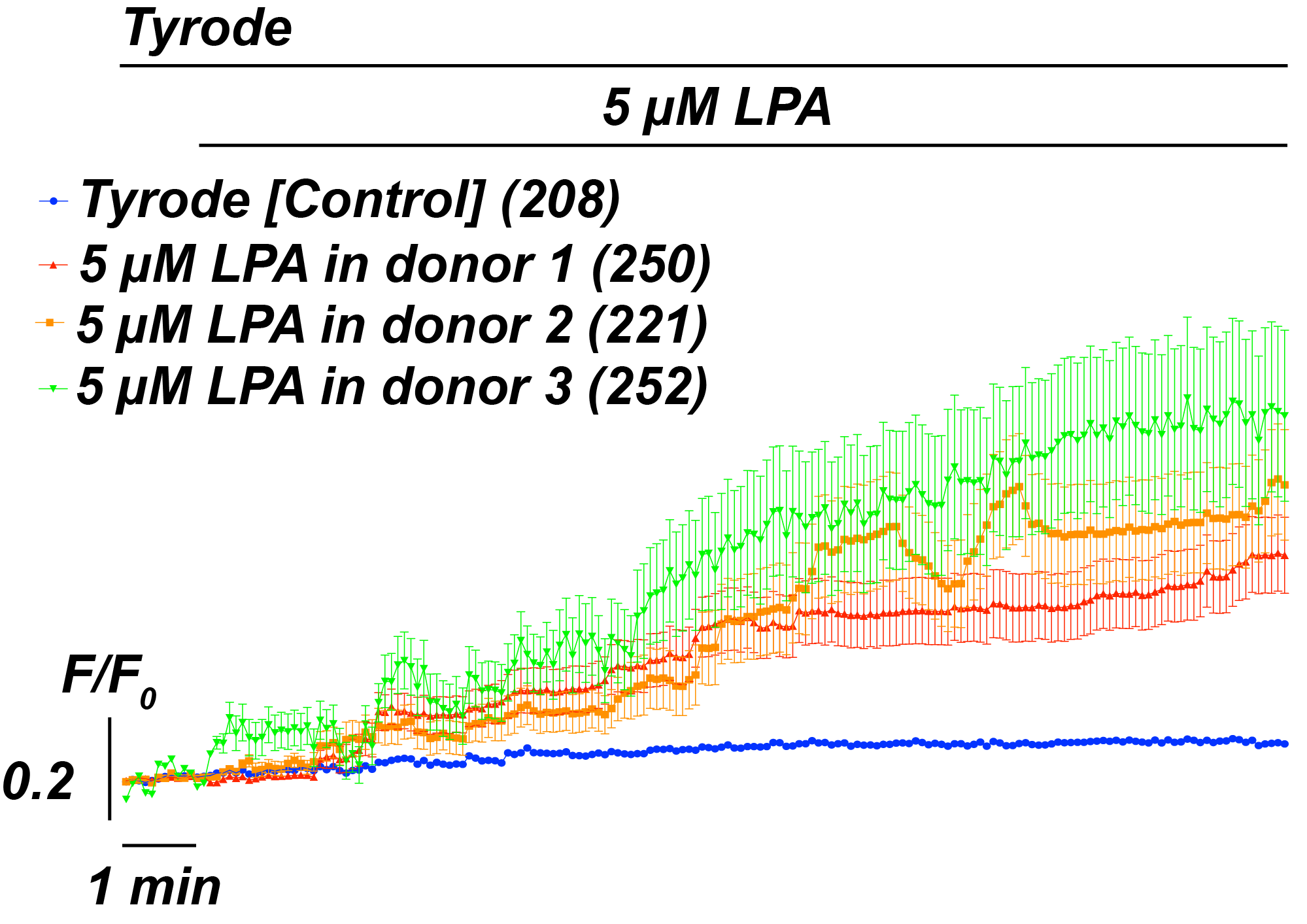

Supplement: Figure S3 — Average Ca2+ signals after 5 µM LPA stimulation for 3 individual healthy donors indicating the degree of inter-individual variations. (TIF) [file pone.0067697.s003.tif]

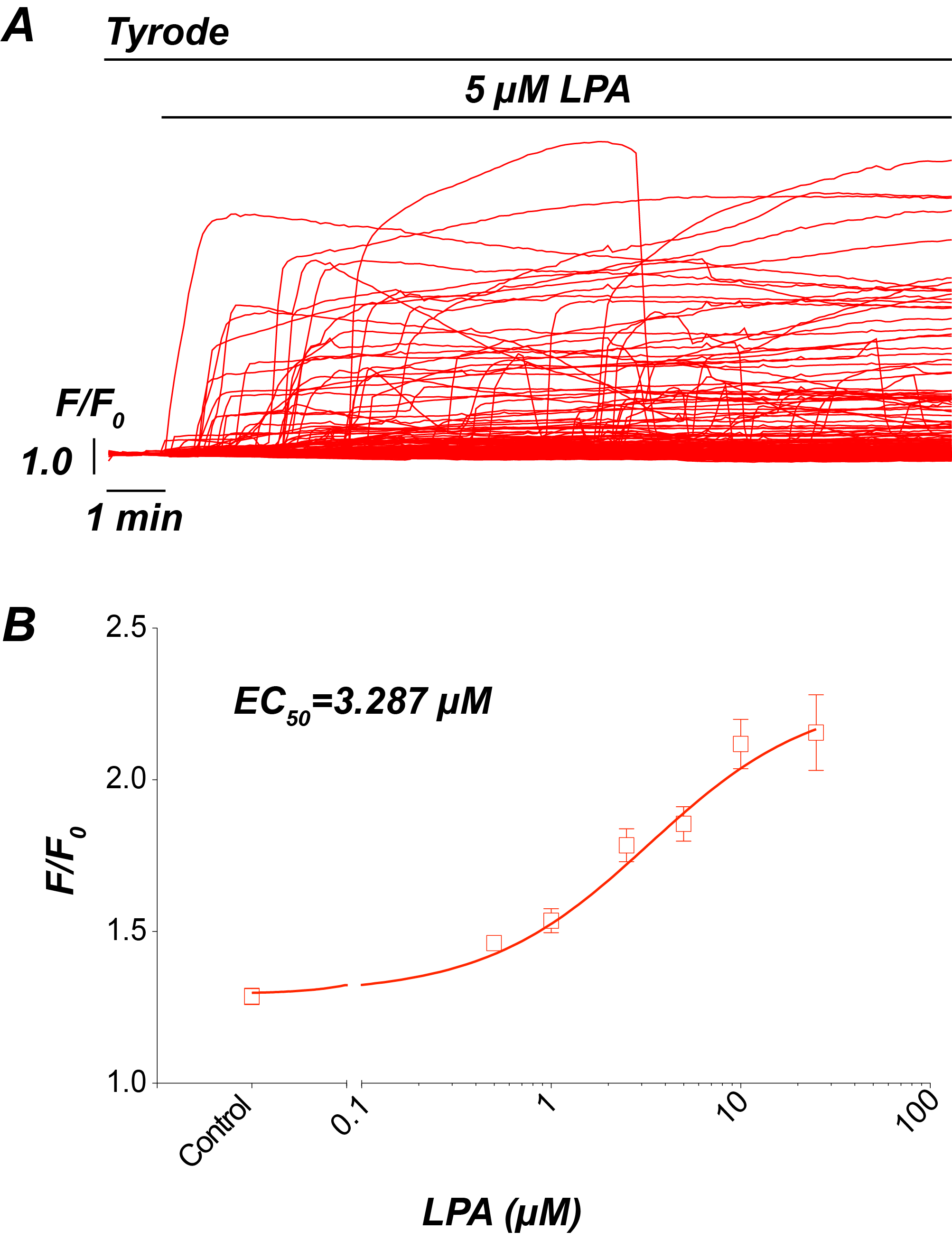

Supplement: Figure S4 — LPA stimulation of mouse RBCs. (A) Single-cell fluorescence response of mouse RBCs after stimulation with 5 µM LPA. (B) Dose response relationship of the LPA concentration with a calculated EC50 of 3.3 µM, which is close to the value for human RBCs (5.0 µM) (compare to Figure 1). (TIF) [file pone.0067697.s004.tif]

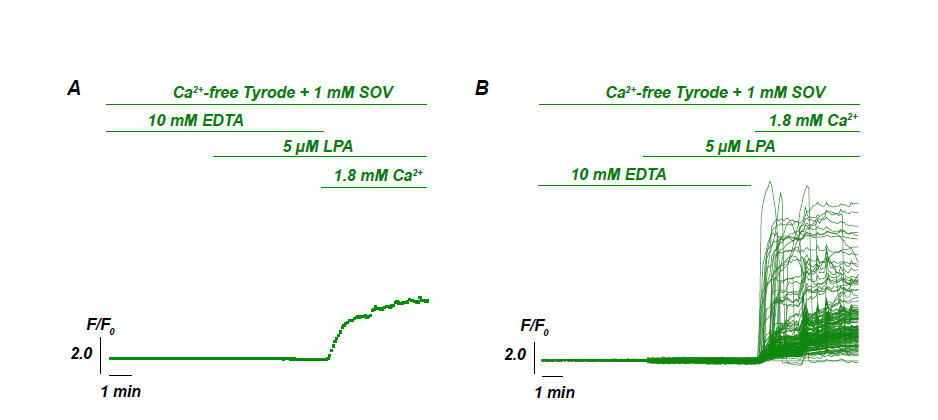

Supplement: Figure S5 — Panel (A) depicts the attempt to “synchronize” the cells by applying a three step protocol starting with the application of a Ca2+ free solution and inhibition of the Ca2+ pump with sodium orthovanadate (SOV). Then, the RBCs were stimulated with LPA for 5 min, and Ca2+ (1.8 mM) was added, leading to a synchronized cell response. (B) Single-cell traces show [same data as in (A)] that the cells still respond variably to the Ca2+ readdition. (TIF) [file pone.0067697.s005.tif]
